# The TLR3 L412F polymorphism prevents TLR3-mediated tumor cell death induction in pediatric sarcomas

**DOI:** 10.1038/s41420-023-01513-y

**Published:** 2023-07-07

**Authors:** Joseph Bisaccia, Swann Meyer, Adrien Bertrand-Chapel, Quentin Hecquet, Virginie Barbet, Bastien Kaniewski, Sophie Léon, Nicolas Gadot, Isabelle Rochet, Iveta Fajnorova, Pierre Leblond, Martine Cordier-Bussat, Nadège Corradini, Alexandre Vasiljevic, Marc Billaud, Cécile Picard, Laura Broutier, Cindy Gallerne, Aurélie Dutour, Jean-Yves Blay, Marie Castets

**Affiliations:** 1 Cell Death and Childhood Cancers Laboratory, LabEx DEV2CAN, Centre de Recherche en Cancérologie de Lyon, INSERM U1052- CNRS UMR5286, Université Claude Bernard de Lyon, Centre Léon Bérard, Institut Convergence Plascan, Lyon, France; 2 EX-VIVO Platform, Université de Lyon, Université Claude Bernard de Lyon, INSERM 1052, CNRS 5286, Centre Léon Bérard, Centre de recherche en cancérologie de Lyon (CRCL), Lyon, France; 3 Anatomopathology Research Platform, Department of Translational Research and Innovation, Centre Léon Bérard, Lyon, France; 4 Department of Pathology, Hôpital Femme-Mère-Enfant, Hospices Civils de Lyon, Université de Lyon, Lyon, France; 5 Department of Pediatric Oncology, Institut d’hématologie et d’oncologie pédiatrique, Centre Léon Bérard, Lyon, France; 6 Department of Translational Research in Paediatric Oncology, Centre Léon Bérard, Lyon, France; 7 Department of Pediatric Oncology, Institut d’Hématologie et d’Oncologie Pédiatrique, Centre Léon Bérard, Lyon, France

**Keywords:** Cancer, Cell death

## Abstract

Toll-like receptor 3 (TLR3) is a pattern recognition receptor mainly known for its role in innate immune response to infection. Indeed, binding of double-stranded RNA (dsRNA) to TLR3 triggers a pro-inflammatory cascade leading to cytokine release and immune cell activation. Its anti-tumoral potential has emerged progressively, associated with a direct impact on tumor cell death induction and with an indirect action on immune system reactivation. Accordingly, TLR3 agonists are currently being tested in clinical trials for several adult cancers. Meanwhile, TLR3 variants have been linked to auto-immune disorders, and as risk factors of viral infection and cancers. However, aside from neuroblastoma, TLR3 role in childhood cancers has not been evaluated. Here, by integrating public transcriptomic data of pediatric tumors, we unveil that high TLR3 expression is largely associated with a better prognosis in childhood sarcomas. Using osteosarcomas and rhabdomyosarcomas as models, we show that TLR3 efficiently drives tumor cell death in vitro and induces tumor regression in vivo. Interestingly, this anti-tumoral effect was lost in cells expressing the homozygous TLR3 L412F polymorphism, which is enriched in a rhabdomyosarcomas cohort. Thus, our results demonstrate the therapeutic potential associated with the targeting of TLR3 in pediatric sarcomas, but also the need to stratify patients eligible for this clinical approach with respect to the TLR3 variants expressed.

## Introduction

Sarcomas encompass a highly heterogeneous group of tumors of mesenchymal origin. They represent less than 1% of adult cancers, but approximately 15% of pediatric ones [1]. More than 100 nosological forms have recently been listed in the last version of the WHO classification [2], with two main groups: Soft-Tissue Sarcomas (STS) and Bone Sarcomas.

While Rhabdomyosarcomas (RMS) account for 50–60% of pediatric STS, Osteosarcomas (OS) are the most frequent primary cancer of bone in children and adolescents [3]. RMS show similarities with skeletal muscle in their histology and gene expression profile, but are a complex group comprising several malignant subtypes [4]. Part of this complexity was elucidated by the identification of the PAX3/7-FOXO1 translocation, which is characteristic of the high-risk subgroup of RMS, called fusion-positive RMS (FP-RMS) [5]. The remaining RMS affecting children and adolescents correspond primarily to the so-called embryonal histology, and is largely defined by default as the fusion-negative group (FN-RMS) from a molecular perspective [6]. OS have an incidence of 4–5 cases per million per year [3]. They occur mainly in the long bones of the limbs, near the metaphyseal growth plate. Tumor development is characterized by the formation of immature bone, also called osteoid tissue, and is associated with para-tumoral osteolysis. OS often present high local aggressiveness and rapid metastasizing potential [7]. Tumors exhibit complex rearrangements, including structural and numerical aberrations, and high genomic heterogeneity between cells. Only a few conserved genetic changes that may constitute effective therapeutic targets have been identified [8]. In RMS and OS, treatments generally combine chemotherapy and secondary surgery, often associated with radiotherapy for high-risk patients. Despite these aggressive therapies, the 5-year survival rate ranges from 60 to 80% for RMS patients with localized tumors, and the majority of modern series report a 3-year disease-free survival of 60–70% in OS. However, the overall 5-year survival rate does not exceed 25% in case of metastases [9]. In addition, such heavy treatments are not devoid of long-term side-effects, especially harmful during childhood. Lastly, despite the implementation of several international clinical trials, patient outcome has not improved significantly over the last 20 years, further emphasizing the importance of dissecting their molecular etiology.

Resistance to cell death plays a key role in the early stages of tumorigenesis, as well as in constitutive or acquired resistance to treatments [10]. While the exploration of therapeutic opportunities associated with the activation of apoptotic pathways has been largely unsuccessful, new levers have recently emerged with the identification of alternative programmed cell death pathways, and the demonstration of plasticity between these different signaling cascades [11, 12]. Along this line, we have recently shown the therapeutic potential of targeting the Toll-Like Receptor 3 (TLR3) to trigger cell death in neuroblastoma [11].

TLR3 is a pattern recognition receptor that is activated by the binding of double-stranded RNA (dsRNA). It has a physiological role in innate immune system activation, notably in response to viral infection, by triggering IRF3 (Interferon Regulatory Factor 3) and NF-κB signaling, thereby triggering an inflammatory response.

However, TLR3 was also shown to function as a death receptor in cancer cells when engaged with dsRNA [13]. Of note, the death signal transduction associated with TLR3 activation has a plastic component, since it results in the activation of apoptosis [14], necroptosis [15], or a form of lysosomal death [11], depending on the functional effectors present in cancer cells. Pre-clinical and clinical trials based on the use of synthetic dsRNAs activating TLR3 are currently underway in adult cancers, but could also represent a promising therapeutic strategy in children and adolescents [16]. Identifying TLR3 as a therapeutic target requires the identification of patients who will respond.

Here, we show that the level of TLR3 expression has a good predictive value in childhood sarcomas, and notably in FP-RMS and OS. Our results confirm the therapeutic potential of inducing TLR3, since its triggering by IFN-1, and its subsequent activation by dsRNA effectively lead to OS and RMS tumor cell death in vitro and in vivo. Importantly, this effect is not observed in cells expressing the homozygous TLR3 L412F polymorphism. Replacement of one of the two L412F alleles in tumor cells by a CRISPR-Cas9 Knock-In approach is sufficient to restore TLR3-mediated cell death. Finally, we found that the homozygous L412F polymorphism was present at a significantly higher frequency in FN-RMS tumors than in the general population, suggesting that it may constitute a risk factor for the occurrence of these tumors and a predictive marker of response to TLR3 treatment.

## Results

### TLR3 expression levels correlate with favorable prognosis in patients

**Fig. 1:**
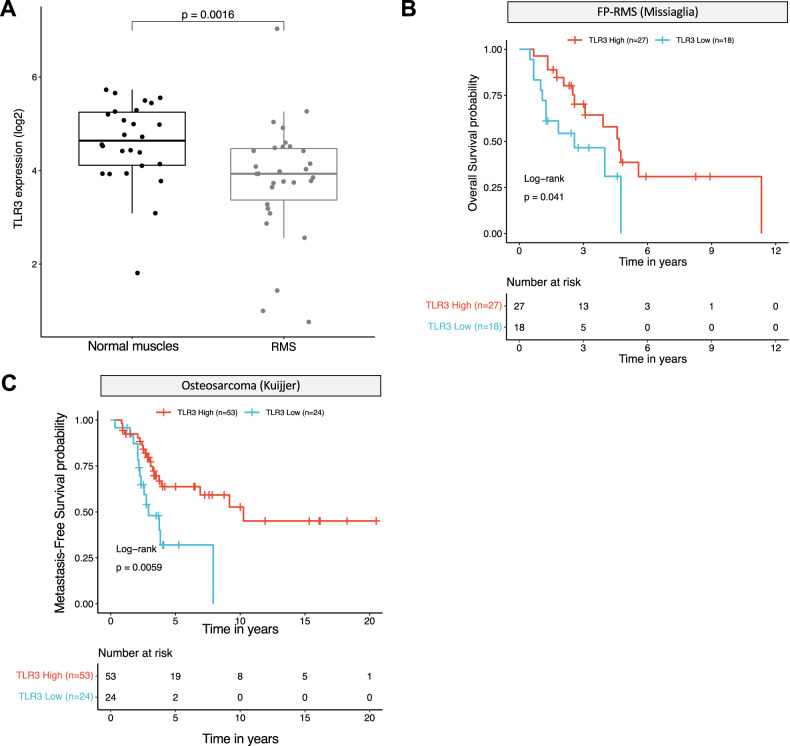
High TLR3 expression is associated with a better prognosis in pediatric and AYA sarcomas. **A** TLR3 expression is decreased in rhabdomyosarcomas (RMS) compared to their non-tumoral counterpart, i.e. normal skeletal muscles (*p*-value = 0.0016, unpaired two-sided Wilcoxon test). Publicly available transcriptomic data from Schäfer and Welle’s cohort were downloaded from R^2^ cancer. **B** TLR3 expression is associated to a better overall survival probability in pediatric Fusion Positive-RMS samples (*p*-value = 0.041, log-rank test). Publicly available E-TABM-1202 transcriptomic data were downloaded from ArrayExpress. **C** TLR3 expression is associated with a better metastasis-free survival probability in a cohort of pediatric and AYA osteosarcomas (*p*-value = 0.0059, log-rank test). Publicly available transcriptomic data from Kuijjer’s cohort were downloaded from R^2^ cancer.

The prognostic value of TLR3 expression has been reported in several cancers [17]. A high level of expression of this receptor has been associated with good [18] and poor [19] prognosis, depending either on the propensity of TLR3 to initiate cell death or to drive inflammation. Using publicly available transcriptomic datasets, we analyzed the prognostic impact of TLR3 expression in cohorts of patients with sarcomas. As shown in Fig. 1A, TLR3 expression level is lower in pediatric RMS than in their non-tumoral skeletal muscle counterpart, which has a rather low level of expression of the receptor compared to other normal tissues (Supplementary Fig. S1A). We then stratified RMS according to their fusion-status. In pediatric FP-RMS, the level of TLR3 expression is positively correlated with patient prognosis in two independent cohorts (Fig. 1B and Supplementary Fig. S1B), and the same trend is observed in FN-RMS (Supplementary Fig. S1C), although not significantly probably due to their overall better outcome. Similarly, the survival rate of patients with high TLR3 mRNA levels is significantly higher in leiomyosarcomas and in a cohort of Ewing sarcoma tumors from children, adolescent and young adults (AYA) (Supplementary Fig. S1D, E). Lastly, in OS, pediatric and AYA patients with a high TLR3 expression level present a better metastasis-free survival likelihood (Fig. 1C).

Hence, TLR3 expression is largely associated with a favorable outcome notably in pediatric and AYA sarcomas, suggesting that it may function mostly as a death receptor in those tumor cells.

### Activation of TLR3 by synthetic dsRNA can trigger RMS and OS tumor cell death in vitro and in vivo

**Fig. 2:**
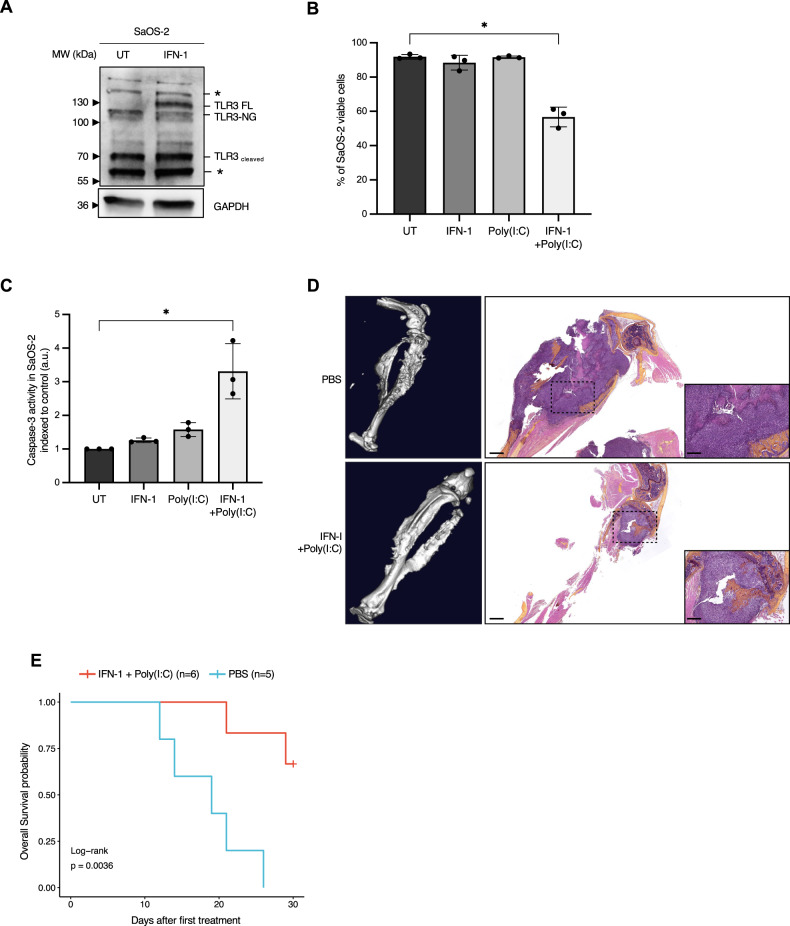
TLR3 can mediate sarcoma cell death in vitro and in vivo. **A** Treatment with IFN-1 is sufficient to restore TLR3 expression in SaOS-2 osteosarcoma (OS) cells. Immunoblot against TLR3 was performed on cell lysates, 24 h after IFN-1 treatment. GAPDH is used as a loading control. One representative image out of 3 independent experiments is shown. FL Full Length, NG Non-Glycosylated. Asterisks denote unspecific bands. **B** Activation of TLR3 by Poly(I:C) triggers SaOS-2 cell death in vitro. Viability of SaOS-2 osteosarcoma cells treated or not with IFN-1 and/or Poly(I:C) for 72 h was analyzed by cytometry (DAPI/Acridine Orange). Mean values ± s.t.d are represented (*n* = 3, *p-value = 0.05). **C** Activation of TLR3 by Poly(I:C) leads to an increase in Caspase-3 activity in SaOS-2 cells in vitro. Mean values ± s.t.d of caspase-3 activity quantified according to the fluorescence emitted by cleavage of a DEVD-AFC peptide are represented (*n* = 3, *p-value = 0.05). **D**, **E** Activation of TLR3 slows osteosarcoma progression in vivo. **D** Left panel. CT-Scan imaging of mice tibia/fibula bones, 15 days after SaOS-2 osteosarcoma cell engraftment. One representative image is shown for each group. Right panel. Representative images of Hematoxylin Phloxin and Safran staining showing dense, poorly differentiated, high-grade cells in a PBS treated mice (*n* = 5), and a smaller tumor in an IFN-1+Poly(I:C) treated animal (*n* = 6). Black scales: 1 mm (low magnification)/400 μm (high magnification). Mouse overall survival is represented as (**E**) a Kaplan–Meier curve (*p*-value = 0.0036, log-rank test).

We then aimed at defining whether TLR3 activation by synthetic dsRNA, namely Poly(I:C), was sufficient to trigger sarcoma cell death. Since TLR3 expression levels are low in all RMS and OS cell lines available, and considering the fact that TLR3 was previously shown to be an IFN-1 response gene [20], cells were pre-treated with IFN-1 prior to exposure to Poly(I:C) to increase TLR3 expression. As shown in Fig. 2A and Supplementary Fig. S2A, IFN-1 induces a robust increase in TLR3 production in SaOS-2 and MNNG/HOS OS cell lines. The same result was obtained using a home-made FN-RMS cell line derived from a patient biopsy at diagnosis, thereafter referred to as RMS-CLB1 (Supplementary Fig. S2B). Consistent with its function to induce cell death, exposure of IFN-1-treated SaOS-2 cells to Poly(I:C) triggers tumor cell death (Fig. 2B). SaOS-2 cell death induction is associated with an increase in the activity of the pro-apoptotic cysteine protease, Caspase-3 (Fig. 2C), suggesting that TLR3 activation drives apoptosis of these OS cells. Similarly, activation of TLR3 by Poly(I:C) triggers a significant increase in tumor cell death, both in MNNG/HOS (Supplementary Fig. S2C) and RMS-CLB1 (Supplementary Fig. S2D). To extend these findings, we designed a murine orthotopic model of OS, by grafting SaOS-2 cells in the tibia of immunocompromised mice. Following tumor uptake and validation by CT-scan, mice were treated with a combination of IFN-1 and Poly(I:C), or with PBS as a control, and imaged until one of the endpoints described in the Material and Methods section was reached. In the control group, CT scans of tumor-bearing paws show “spindle-like” ossifications outside the tibia and an aggressive periosteal reaction (sunburst structures, Fig. 2D, upper panel). These OS structures are less pronounced in the treated group of mice, with the cortical structure of the bones being largely preserved in those animals (Fig. 2D, bottom panel). Accordingly, the survival of mice is significantly higher in the treated group than in the control one (Fig. 2E; log-rank test, *p* = 0.03).

Altogether, these data confirm that TLR3 acts as a death receptor both in vitro and in pre-clinical xenograft models to trigger RMS and OS cell death.

### L412F polymorphism abolishes cell death function of TLR3 in RMS cells

**Fig. 3:**
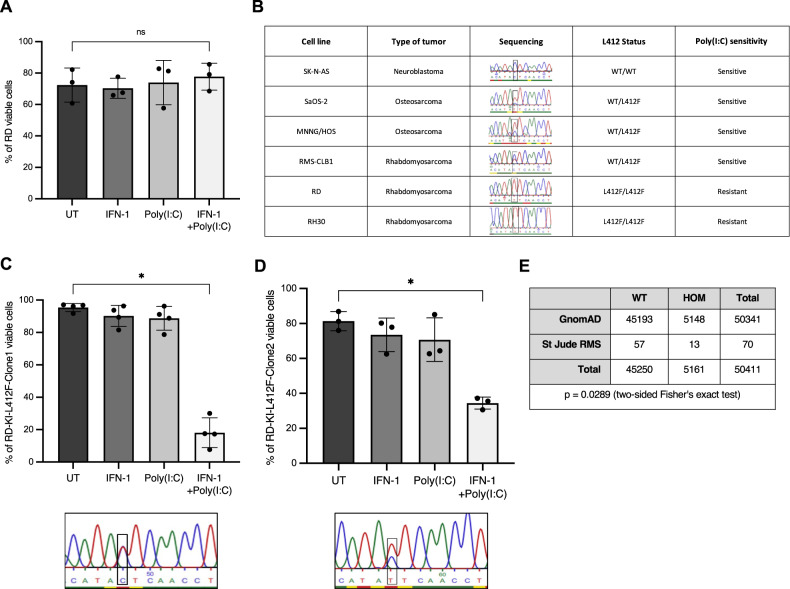
L412F polymorphism prevents TLR3-induced cell death. **A** Treatment with Poly(I:C) does not trigger RD cell death in vitro. Viability of RD rhabdomyosarcoma (RMS) cells treated or not with IFN-1 and/or Poly(I:C) for 72 h was analyzed by cytometry (DAPI/Acridine Orange). Mean values ± s.t.d are represented (*n* = 3, *p*-value = 0.35). **B** Homozygous status for the L412F variant of TLR3 correlates with Poly(I:C) insensitivity in pediatric Osteosarcoma (SaOS-2, MNNG/HOS), Rhabdomyosarcoma (RD, RH30, RMS-CLB1) and Neuroblastoma (SK-N-AS) cell lines. **C**, **D** Expression of one TLR3 L412 allele is sufficient to restore sensitivity of RD cells to Poly(I:C). L412 allele was inserted in two RD clones using a CRISPR-Cas9 Knock-In strategy, respectively termed RD-KI-L412F-Clone1 (**C**) and RD-KI-L412F-Clone2 (**D**). Cells were treated with IFN-1 and/or Poly(I:C) for 72 h, using DAPI/Acridine Orange. Mean values ± s.t.d are represented (RD-KI-L412F-Clone1, *n* = 4, *p-value = 0.0143; RD-KI-L412F-Clone2, *n* = 3, *p-value = 0.05). **E** Percentage of homozygous individuals is higher in a cohort of children and AYA with RMS than in the general population. TLR3 L412 variant status was defined in the St Jude RMS cohort relative to the GnomAD v3.1.2 cohort of healthy individuals (*p-value = 0.0289, two-tailed Fisher’s exact test).

However, when screening sarcoma cells to define the role of TLR3, we observed that a fraction of RMS cell lines were resistant to IFN-1/Poly(I:C) treatment. In RD cells, TLR3 expression is well induced by IFN-1 (Supplementary Fig. S3A). However, as shown in Fig. 3A, this treatment has no effect on the FN-RMS RD cell line, neither in terms of death induction (Fig. 3A) nor Caspase-3 activation (Supplementary Fig. S3B). Loss of inflammatory [18] or death activity [19] of the TLR3 receptor has previously been correlated with the L412F polymorphism (rs3775291), which has an allelic frequency of approximately 24% in the general population [20]. We thus assessed the allelic status of TLR3 in several childhood cancer cell lines. As shown in Fig. 3B, all cell lines sensitive to TLR3 activation express the L412 reference allele either at the homozygous or heterozygous state. Reciprocally, the RD resistant cell line, as well as the FP-RMS RH30 line, express the L412F polymorphism homozygously. Consistently, while TLR3 expression is also significantly increased by IFN-1 in those cells (Supplementary Fig. S3C), it fails to engage cell death in response to Poly(I:C) in RH30 cells (Supplementary Fig. S3D). Of note, transient expression of the L412 allele of TLR3 in RD cells is sufficient to trigger cell death upon Poly(I:C) treatment (Supplementary Fig. S3E, F). Since expression of one copy of the reference L412 allele seems to be sufficient to restore TLR3 function, we used a CRISPR-Cas9 Knock-In strategy to correct one of the two F412 alleles in the RD cell line. We obtained two clones, in which we successfully replaced one of the two F412 alleles by the L412 one at the endogenous locus. As shown in Fig. 3C, D, introduction of the L412 correction in both RD-KI-L412F clones (clones 1 and 2) is sufficient to restore cell death in response to Poly(I:C).

We then questioned the clinical value of L412F status in RMS patients. Because of its high population frequency this variant is filtered out during the processing steps of somatic variant calling, making it difficult to analyze from public databases. However, we were able to perform an analysis of its frequency in patients with FN-RMS, thanks to the raw data available on the St. Jude cloud. As shown in Fig. 3E, the number of FN-RMS patients presenting the homozygous L412F allele was significantly higher than expected compared to the general population (two-sided Fisher’s exact test, *p* = 0.0289).

Collectively, these results indicate that the homozygous expression of the L412F allele blocks TLR3 death receptor function in pediatric RMS, and could be associated with an increased risk of occurrence of these cancers.

## Discussion

In addition to its well-characterized role in the activation of the immune response via the IRF3/NF-KB pathway [21, 22], TLR3 is emerging as a potential anti-tumor target in cancers, due to its ability to induce different death signals, depending on the cellular context [11]. Targeting TLR3 could then have two complementary anti-tumor effects, directly by triggering death signaling pathways in tumor cells, and indirectly, by stimulating anti-tumor immunity through cytokine release.

Until now, the clinical value of TLR3 has mainly been studied in adult cancers, and mostly in carcinomas [23], although the beneficial effect of a high level of TLR3 expression has also been established in Neuroblastomas [24]. The data presented here clearly demonstrate that the clinical potential associated with TLR3 activation is not restricted to the adult population but could also be a promising avenue for the treatment of children and adolescent’s cancers.

By integrating transcriptomic data, we established here the prognostic value of TLR3 expression in several pediatric, adolescent and young adult sarcomas. The implementation of this parameter at the time of diagnosis could be useful to improve the risk stratification of young patients with sarcomas, considering the complexity and heterogeneity of these cancers. Moreover, our results reveal that it is possible to restore TLR3 expression in aggressive tumor cells via IFN-1 pre-treatment, and that the addition of Poly(I:C) dsRNA is then sufficient to trigger TLR3-induced cell death in vitro and in vivo, notably in osteosarcoma models. IFN-1 was already included in a randomized clinical trial in combination with chemotherapy to compare its benefit in treating patients with osteosarcoma (NCT00134030). Although it was not associated with a substantial advantage in this study, it however shows the feasibility of using a combination of IFN-1 and TLR3 agonists, since such compounds are currently evaluated in some clinical trials [23].

However, our results highlight the need to precisely define the pool of patients eligible for this therapeutic approach, given the existence of polymorphisms that alter the functionality of this receptor. Higher risk of infections by CMV, HSV-1 or HSV-2 [25–27], as well as a higher susceptibility to develop asthma were notably attributed to the expression of L412F variant in cohorts of children [28]. More recently, the L412F polymorphism was also linked to the severity of SARS-CoV-2 infection [29], as well as with an increased risk of developing colorectal and oral cancers [30, 31]. These observations were linked to the fact that replacement of the lysine by a phenylalanine at position 412 induces a disruption of the structure of the TLR3 ectodomain, decreasing the receptor-ligand binding capacity and preventing the glycosylation of neighboring residues, which is necessary for TLR3 signal transduction [32–34]. Accordingly, the induction of the NF-κB or of the autophagic pathways are significantly reduced when the variant is expressed [26]. *Vice et versa*, the expression of the L412F polymorphism is associated with a reduced risk of macular degeneration, which was shown to result from a decreased propensity of this TLR3 allele to drive retinal cell apoptosis [35]. Our data reconcile these two series of observations, by showing that the L412F variant plays an oncogenic role not only by altering the anti-tumor immune response, but also by blocking the transduction of the death signal in tumor cells.

We also found that the incidence of the rs3775291 polymorphism may be higher in FN-RMS than in the general population. Although this finding needs to be validated using independent cohorts of patients, it suggests that blockage of TLR3 functionality might also be considered as a risk factor for pediatric sarcomas. Considering the therapeutic potential of TLR3, further analyses are required to define the clinical value of this polymorphism, whether it may prevent the use of TLR3 as a therapeutic target or constitute a genetic biomarker of a poorer outcome with resistance to treatment.

## Material and methods

### Cell culture

In vitro studies were conducted using human RD FN-RMS cells (ATCC), RH30 FP-RMS cells (DSMZ), SK-N-AS neuroblastoma cells (ATCC), and SaOS-2 and MNNG/HOS OS cells (ATCC). All cell lines were routinely maintained under standard conditions (37°C and 5% CO_2_ in a humidified incubator) in Dulbecco’s Modified Eagle’s Medium (DMEM), except RH30 cells that were cultured in Roswell Park Memorial Institute (RPMI). All media were supplemented with 10% Fetal Bovine Serum (FBS) (CVFSVF00-01, Eurobio Scientific) and 1% of Penicillin and Streptomycin (14140-122, Thermo Fisher). The RMS-CLB1 primary cell line was derived from a patient biopsy at relapse, and cultured as described previously [36].

All cells were monthly tested to ensure a mycoplasma-free condition.

### Cell death assays

100,000 cells were seeded in 6-wells plates in normal growth medium for 72 h. To induce TLR3 expression, cells were then pre-treated with 1000 units/mL Universal Type I IFN-1 Protein (1200-2, BioTechne). Alternatively, TLR3 (pcDNA3-TLR3-CFP plasmid #13641 from Addgene, gift from Doug Golenbock; http://n2t.net/addgene:13641; RRID:Addgene_13641) was transiently expressed in RD cells using jetPRIME reagent (Polyplus transfection) following manufacturer’s instructions (2:2 DNA/jetPRIME® ratio). After 24 h, cells were starved in their respective medium containing 0.1% FBS to sensitize cells to death as previously described (Terra et al., 2011, Diabetologia; Castets et al., 2009, Dev Cell; Sun et al., 2011, J Biol Chem), and treated or not with IFN-1 1 000 Units/mL and/or 10 µg/mL Poly(I:C) (tlrl-pic, InvivoGen).

Viability was estimated at 24, 48 and 72 h post-treatment, using an Acridine Orange/DAPI double staining on a NucleoCounter NC-3000 (Chemometec, Allerød, Denmark) according to the manufacturer’s instructions (Kit 910-3013, Chemometec).

For Caspase-3 assay, cells were collected 24 h after treatment and lysed in 55 µL of dedicated buffer according to the manufacturer’s instructions (K105, BioVision). 50 µL of cell lysates were mixed with an equal volume of reaction buffer. Fluorescence resulting from DEVD-AFC cleavage was followed and quantified on a Tecan Infinite® M1000 PRO.

### Western blot analyses

100,000 cells were seeded in 6-wells plates in normal growth medium for 24 h. To induce TLR3 expression, cells were then pre-treated with 1000 units/mL Universal Type I IFN-1 Protein (11200-2, BioTechne).

24 h later, cells were collected and lysed in 30 µL of RIPA buffer (50 mM Tris-HCl pH = 8.0, 150 mM NaCl, 1% Triton-X100, 0.1% SDS, 0.5% deoxycholic acid, with protease and phosphatase inhibitors (78430, ThermoFisher)). Protein extracts were then analyzed by immunoblot. Briefly, proteins were quantified using a BCA assay kit (23225, ThermoFisher), then loaded onto 7.5% or 10% SDS polyacrylamide gels (4561026, 4561023; Biorad) and blotted onto nitrocellulose sheets (1704159; BioRad) using the TurboBlot technology (BioRad). Membranes were blocked with 5% BSA in TBS/0.1% Tween 20 for 1 h and then incubated overnight with anti-TLR3 (1/1000, 6961, Cell Signaling) or anti-GAPDH (1/1000, 5174, Cell Signaling). After three washes with TBS, membranes were incubated with the appropriate HRP-conjugated secondary antibody (1/20 000, Jackson ImmunoResearch) for 1 h. Detection was performed using the ECL Prime (RPN2236, Cytiva). Membranes were imaged on the ChemiDoc Touch Imaging System (BioRad). Uncropped Blots are available as a separate Supplemental Material file.

### Amplification and sequencing of the L412 TLR3 region

Genomic DNA was extracted from samples using DNeasy Blood & Tissue Kit (69504, Qiagen) following the manufacturer’s instructions. The amplification of the region of interest, surrounding the L412F site, was done by PCR using the 5X PCR Firepol Kit (04-12-00115, Solys BioDyne) and TLR3-specific primers (forward primer: 5′-CCAACTCCTTTACAAGTTTGCG-3′ and reverse primer 5′-CCAGGTCAAGTACTTCTAGG-3′). PCR products were purified from an agarose gel using the PCR Clean-up kit (740609, Macherey Nagel). PCR products were then sequenced using the following primer: 5′-CCAACTCCTTTACAAGTTTGCG-3′.

### Correction of L412F polymorphism by CRISPR-Cas9

To perform a single-base substitution from an annotated polymorphism to the reference L412 allele in RD cells, we applied a genome editing strategy based on the use of a mix composed of a crRNA (Alt-R®CRISPR-Cas9 crRNA; 5′- GGTTAGGTTGAATATGTGTA -3′), a tracrRNA (Alt-R®CRISPR-Cas9 tracrRNA, ATTO™550, 1075927), the nuclease from idtDNA (Alt-R® S.p. HiFi Cas9 Nuclease V3, 1081060) and a plasmid encoding L412 TLR3 allele from Addgene (pcDNA3-TLR3-CFP plasmid #13641, gift from Doug Golenbock; http://n2t.net/addgene:13641; RRID:Addgene_13641). sgRNA were found with CRISPOR program (http://crispor.tefor.net/) [35]. 3 µL of 200 µM of crRNA and tracrRNA were annealed by heat at 95 °C for 5 min, then cooled at room temperature for 30 min, to allow complex formation. 5 µL of the 100 µM newly-formed complex was then mixed with 4 µL of the electroporation enhancer at 61 µM (Alt-R® Cas9 Electroporation Enhancer, 1075916). 1 million RD cells cultured for 48 h into normal growth medium and resuspended into 90 µL of Opti-MEM medium (31985070 ThermoFisher) supplemented with 100 µM of Rock inhibitor (Y-27632, Sigma-Aldrich) were then electroporated at 175 V 5 ms with 10 µL of RNP complex, electroporation enhancer solution and 5 µg of plasmid using the NEPA21 technology (CU500 system, EC-002S cuvettes, NepaGene). Cells were then allowed to recover in 1 mL of normal growth medium. 48 h after electroporation, ATTO™550 positive cells, which have incorporated the RNP complexes, were sorted by cytometry (BD FACS Aria III) and seeded in a 96-well plate as a single colony. Colonies were cultured and screened by sequencing following the method explained previously.

### Orthotopic xenografts

All experiments were performed in accordance with the regulations for animals used for scientific purposes governed by the European Directive 2010/63/EU. Protocols were validated by the local Animal Ethics Evaluation Committee (CECCAPP: C2EA-15) and authorized by the French Ministry of Education and Research (Authorization #29885).

Five-week-old female BALB/c-NUDE mice were obtained from Janvier labs and housed in sterilized filter-topped cages in a specific pathogen-free (SPF) animal facility P-PAC platform at the Cancer Research Center of Lyon (CRCL), Lyon, France. One week later, 1 M of SaOS-2 cells resuspended in 10 µL of PBS (14190-094, ThermoFisher):Matrigel (10365602, ThermoFisher) (ratio 1:1) were grafted orthotopically in the tibia of mice using a 29 G needle. Tumor engraftment was checked using Vernier Callipers in the first line to evaluate the difference between grafted and non-grafted paws, followed by CT-scan measurement (Quantum FX®, Perkin Elmer). 5–8 days after engraftment, and following tumor uptake validation, IFN-1 (2000 units per gram of mice) and Poly(I:C) (10 µg per gram of mice) were injected intraperitoneally by using PBS (10 µL per gram of mice) as vehicle three times per week. Tumor growth was checked using Vernier Callipers in the first line to evaluate the difference between grafted and non-grafted paws, followed by CT-scan measurement (Quantum FX®, Perkin Elmer), to define experiment’s endpoints, which correspond either to i) swelling of the grafted paw greater than 50% compared to the ungrafted paw of the animal and confirmation of tumor invasion by CT-Scan, or ii) major bone destruction observable on follow-up CT-Scan (*n* = 1 animal in untreated group in our experiment). These endpoints correspond to the time of sacrifice of animals and are used to establish the overall survival curves.

### Patients cohorts and survival analyses

#### Patients cohorts

Publicly available transcriptomic datasets from Kuijjer (osteosarcoma) [37], Dirksen (Ewing sarcoma) [38], TCGA 2022-v32 Sarcoma [39], and Schafer-Welle (RMS and normal muscle) laboratories were downloaded from R2 Cancer. The Kuijjer and TCGA 2022-v32 Sarcoma datasets were filtered to keep only respectively biopsy samples from children and AYA (<35 years old), and leiomyosarcomas (LMS). Missiaglia dataset [6] (accession code ETABM-1202) was downloaded on Array express. The Khan (RMS) dataset was kindly provided by Javed Khan (Center for Cancer Research, National Institutes of Health, Bethesda) [40].

#### Survival analysis

R version 4.2.0 with the packages survminer (v 0.4.9) [41] and survival (v 3.3-1) [42] were used for Kaplan–Meier survival analysis. For the Kuijjer and Dirksen datasets that were downloaded from R2, the optimal TLR3 expression cutoff was determined independently for each subgroup according to the KaplanScan method implemented by R2. For the TCGA 2022-v32 Sarcoma dataset, as online R2 sample annotations differed with the annotations downloaded, the R package maxstat (v 0.7-25) [43] was used to identify the optimal TLR3 expression cutoff. Optimal TLR3 expression cutoff was also inferred using maxstat for Missiaglia and Khan datasets, separately for each fusion status (FP-RMS/FN-RMS). All cutoff values are available in Supplementary Table 1. *p*-values were computed using the log-rank test for all survival analyses.

### Evaluation of TLR3 L412F frequency in pediatric RMS

Reference TLR3 L412 and L412F allele proportions in healthy individuals were retrieved from the GnomAD v 3.1.2 population (https://gnomad.broadinstitute.org/). We used the genomic raw data from St Jude to evaluate the frequency of the L412F allele, both at homozygous and heterozygous states in RMS samples (https://platform.stjude.cloud/) [44]. L412F variants were retrieved from germline gVCF files using tabix (v 1.15.1) [45] based on the following genomic variant: chr4:g.186082920C>T using the GRCh38 genome assembly. When several samples (gVCF files) were available for a subject, only one sample was selected to prevent over- or under-estimating the number of L412F alleles/samples. Enrichment in L412F genotype was assessed using two-tailed Fisher’s exact test.

### Statistics and reproducibility

Statistical significance of differences between groups was evaluated by one-tailed Mann-Whitney tests. Survival analyses were performed using a log-rank test, and enrichment of L412F allele was assessed using two-tailed Fisher’s exact test. *p*-values < 0.05 (*) were considered to be statistically significant. Sample size and replicates are stated in the corresponding figure legends.

## Supplementary information

Supplementary Figures

Supplementary Table 1

Original Data File

## Data Availability

All data generated or analyzed during the study are included in this published article. Requests for material should be made to the corresponding author. Schafer-Welle (RMS and normal muscle), Kuijjer (Osteosarcoma), Dirksen (Ewing sarcoma), TCGA 2022-v32 Sarcoma (Leiomyosarcoma) are available online on the R2 website (https://hgserver1.amc.nl/cgi-bin/r2/main.cgi). The Missiaglia (RMS) dataset is available on ArrayExpress under the identifier E-TABM-1202. The Khan (RMS) dataset can be requested by contacting M.D. Javed Khan (khanjav@mail.nih.gov). Scripts used for the analysis made in this manuscript are available at the following link: 10.5281/zenodo.7852570.
