# Supplementary material for: The TLR3 L412F polymorphism prevents TLR3-mediated tumor cell death induction in pediatric sarcomas
Source: Cell Death Discov. 2023 Jul 7;9:230. doi: 10.1038/s41420-023-01513-y (PMC10326074; doi:10.1038/s41420-023-01513-y)
Supplement: Supplementary file 1 — Supplementary Figures [file 41420_2023_1513_MOESM1_ESM.pdf]

SUPPLEMENTARY FIGURE LEGENDS

Figure S1: TLR3 has a positive prognostic value in several types of sarcomas

(A) Expression level of TLR3 in normal tissues. Publicly available transcriptomic data obtained from GTEX portal. **(B)** Event-free survival probability of FP-RMS samples stratified according to TLR3 expression (*p-value = 0.049, log-rank test). Microarray transcriptomic dataset was provided by Javed Khan's laboratory. **(C)** Overall survival probability of FN-RMS samples stratified according to TLR3 expression (p-value = 0.078, log-rank test). Publicly available E-TABM-1202 transcriptomic data were downloaded from ArrayExpress. **(D)** Overall survival probability of Ewing sarcoma samples stratified according to TLR3 expression (***p-value < 0.0001, log-rank test). Publicly available transcriptomic data from Kuijjer's cohort were downloaded from R^2 cancer. **(E)** Overall survival probability of leiomyosarcomas (TCGA 2022-v32 Sarcoma cohort), stratified according to TLR3 expression (***p-value < 0.0001, log-rank test).

Figure S2: Poly(I:C) treatment drives TLR3-induced cell death in pediatric sarcoma cells.

(A) Immunoblot of TLR3 from MNNG/HOS osteosarcoma cells, treated or not with IFN-1 for 24 h. GAPDH is used as a loading control. One representative image out of 3 independent experiments is shown. Asterisks denote unspecific bands. **(B)** Immunoblot of TLR3 from the patient-derived cell line RMS-CLB1, treated or not with IFN-1 for 24 h. GAPDH is used as a loading control. One representative image out of 3 independent experiments is shown. Asterisks denote unspecific bands. **(C)** Viability of MNNG/HOS osteosarcoma cells treated or not with IFN-1 and/or Poly(I:C) for 72 h was analyzed by cytometry (DAPI/Acridine Orange). Mean values ± s.t.d are represented (n=3, *p-value = 0.05). **(D)** Viability of the patient-derived RMS-CLB1 cell line treated or not with IFN-1 and/or Poly(I:C) for 72 h was analyzed by cytometry (DAPI/Acridine Orange). Mean values ± s.t.d are represented (n=3, *p-value = 0.05).

Figure S3: **RD and RH30 rhabdomyosarcoma cell lines homozygous for the L412F variant of TLR3 are resistant to Poly(I:C).**

(A) Immunoblot of TLR3 from RD rhabdomyosarcoma cells, treated or not with IFN-1 for 24 h. GAPDH is used as a loading control. One representative image out of 3 independent experiments is shown. Asterisks denote unspecific bands. **(B)** Poly(I:C) does not lead to an

increase in Caspase-3 activity in RD cells *in vitro*. Mean values ± s.t.d of caspase-3 activity quantified according to the fluorescence emitted by cleavage of a DEVD-AFC peptide are represented (n = 4, ns p-value = 0.1714). **(C)** Immunoblot of TLR3 from RH30 rhabdomyosarcoma cells, treated or not with IFN-1 for 24 h. GAPDH is used as a loading control. One representative image out of 3 independent experiments is shown. Asterisks denote unspecific bands. **(D)** Viability of RH30 RMS cells treated or not with IFN-1 and/or Poly(I:C) for 72 h was analyzed by cytometry (DAPI/Acridine Orange). Mean values ± s.t.d are represented (n = 3, ns p-value = 0.1). **(E-F)** Transient transfection of the WT allele of TLR3 is sufficient to restore cell death upon Poly(I:C) treatment in RD cells. **(E)** Viability of RD rhabdomyosarcoma cells transfected or not with TLR3 and treated with Poly(I:C) for 48 h was analyzed by cytometry (DAPI/Acridine Orange). Mean values ± s.t.d are represented (n=3, *p-value< 0.05). **(F)** Immunoblot of transfected CFP tagged-TLR3 in RD rhabdomyosarcoma cells. One representative image out of 3 independent experiments is shown. Transfected and endogenous forms of TLR3 are indicated. Asterisks denote unspecific bands. Ctl: RD transfected with an empty vector. Transf. : RD transfected with a CFP-TLR3 L412 encoding vector.

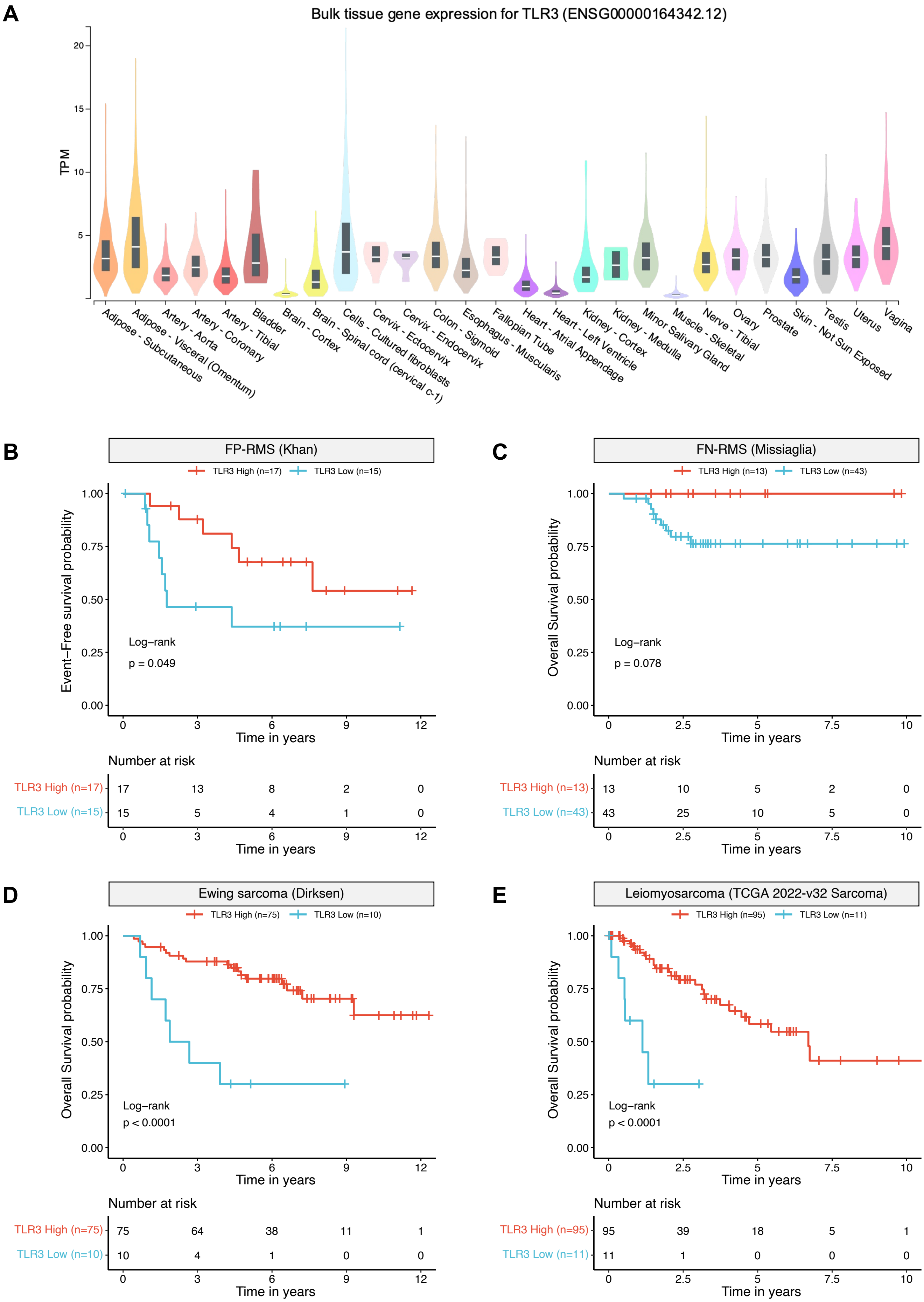
A
Bulk tissue gene expression for TLR3 (ENSG00000164342.12)
TPM
20
15
10
5
Adipose - Subcutaneous
Adipose - Visceral (Omentum)
Artery - Aorta
Artery - Coronary
Artery - Tibial
Bladder
Brain - Cortex
Brain - Spinal cord (cervical c-1)
Cells - Cultured fibroblasts
Cervix - Ectocervix
Cervix - Endocervix
Colon - Sigmoid
Esophagus - Muscularis
Fallopian Tube
Heart - Atrial Appendage
Heart - Left Ventricle
Kidney - Cortex
Kidney - Medulla
Minor Salivary Gland
Muscle - Skeletal
Nerve - Tibial
Ovary
Prostate
Skin - Not Sun Exposed
Testis
Uterus
Vagina
B
FP-RMS (Khan)
TLR3 High (n=17) TLR3 Low (n=15)
Event−Free survival probability
1.00
0.75
0.50
0.25
0.00
Log−rank
p = 0.049
0 3 6 9 12
Time in years
Number at risk
TLR3 High (n=17) 17 13 8 2 0
TLR3 Low (n=15) 15 5 4 1 0
0 3 6 9 12
Time in years
C
FN-RMS (Missiaglia)
TLR3 High (n=13) TLR3 Low (n=43)
Overall Survival probability
1.00
0.75
0.50
0.25
0.00
Log−rank
p = 0.078
0 2.5 5 7.5 10
Time in years
Number at risk
TLR3 High (n=13) 13 10 5 2 0
TLR3 Low (n=43) 43 25 10 5 0
0 2.5 5 7.5 10
Time in years
D
Ewing sarcoma (Dirksen)
TLR3 High (n=75) TLR3 Low (n=10)
Overall Survival probability
1.00
0.75
0.50
0.25
0.00
Log−rank
p < 0.0001
0 3 6 9 12
Time in years
Number at risk
TLR3 High (n=75) 75 64 38 11 1
TLR3 Low (n=10) 10 4 1 0 0
0 3 6 9 12
Time in years
E
Leiomyosarcoma (TCGA 2022-v32 Sarcoma)
TLR3 High (n=95) TLR3 Low (n=11)
Overall Survival probability
1.00
0.75
0.50
0.25
0.00
Log−rank
p < 0.0001
0 2.5 5 7.5 10
Time in years
Number at risk
TLR3 High (n=95) 95 39 18 5 1
TLR3 Low (n=11) 11 1 0 0 0
0 2.5 5 7.5 10
Time in years

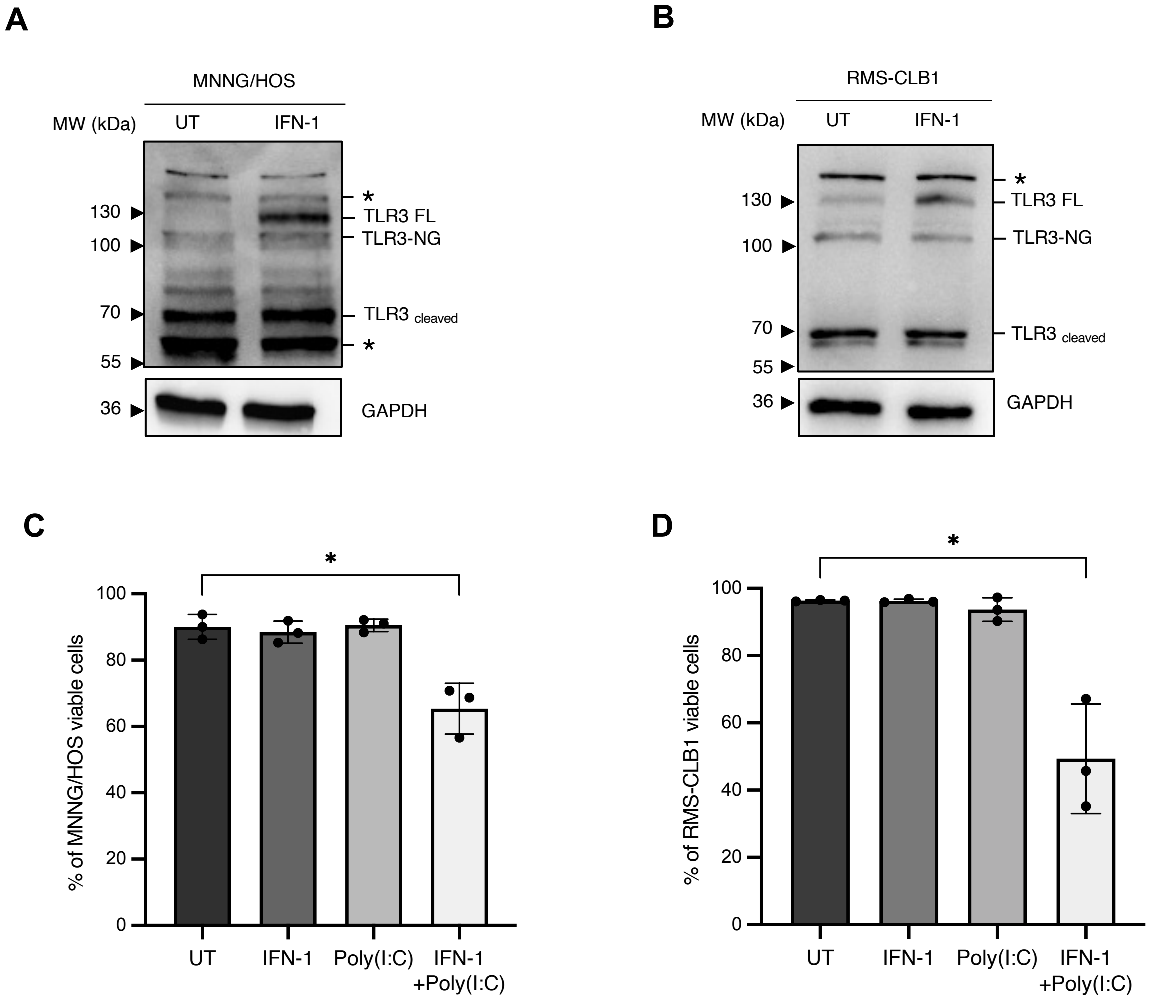
A
MNNG/HOS
MW (kDa)
UT
IFN-1
130
100
70
55
36
*
TLR3 FL
TLR3-NG
TLR3 cleaved
*
GAPDH
B
RMS-CLB1
MW (kDa)
UT
IFN-1
130
100
70
55
36
*
TLR3 FL
TLR3-NG
TLR3 cleaved
GAPDH
C
*
% of MNNG/HOS viable cells
100
80
60
40
20
0
UT
IFN-1
Poly(I:C)
IFN-1
+Poly(I:C)
D
*
% of RMS-CLB1 viable cells
100
80
60
40
20
0
UT
IFN-1
Poly(I:C)
IFN-1
+Poly(I:C)

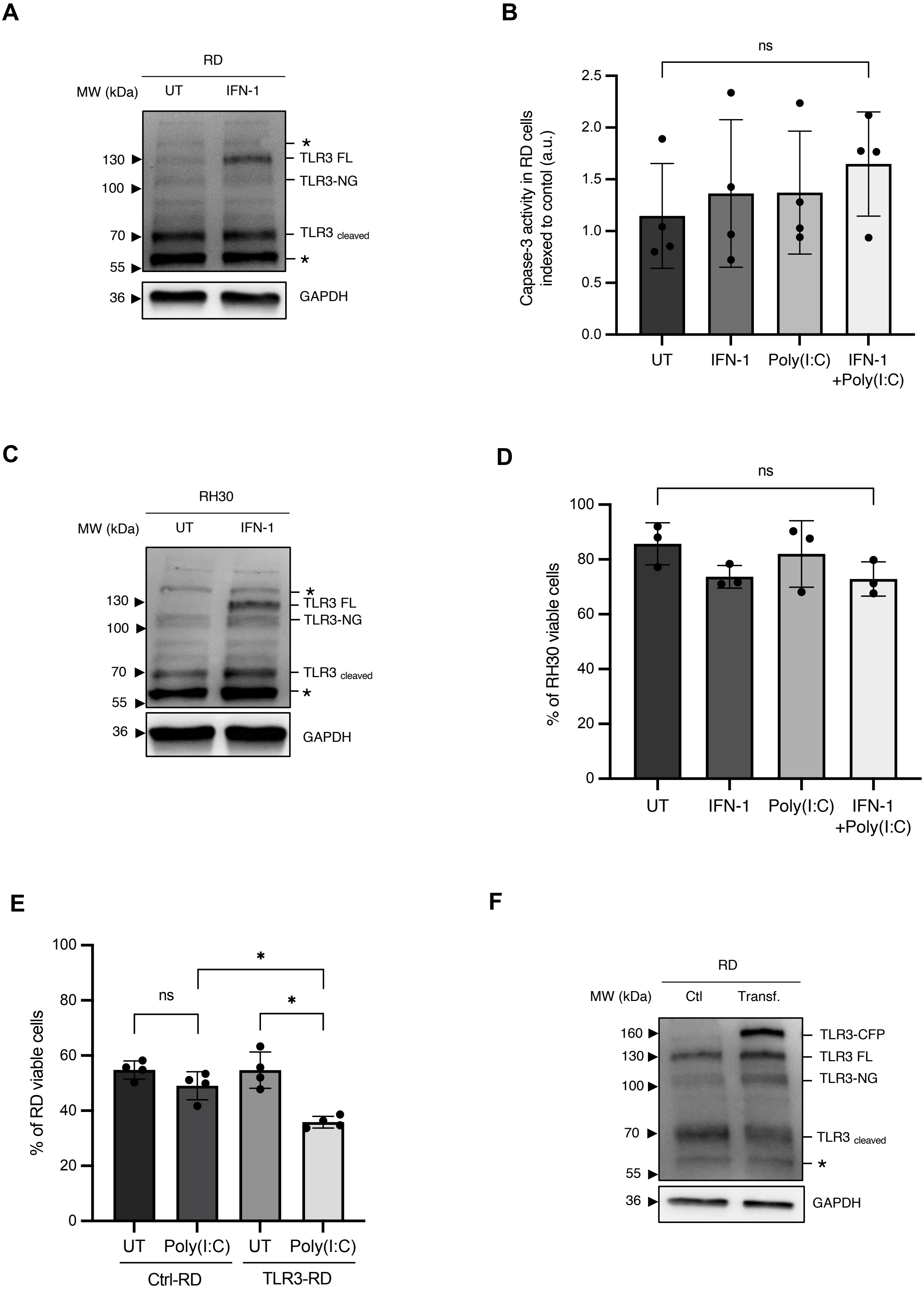
A
RD
MW (kDa)
UT
IFN-1
130
100
70
55
36
*
TLR3 FL
TLR3-NG
TLR3 cleaved
*
GAPDH
B
ns
Capase-3 activity in RD cells indexed to contol (a.u.)
2.5
2.0
1.5
1.0
0.5
0.0
UT
IFN-1
Poly(I:C)
IFN-1 +Poly(I:C)
C
RH30
MW (kDa)
UT
IFN-1
130
100
70
55
36
*
TLR3 FL
TLR3-NG
TLR3 cleaved
*
GAPDH
D
ns
% of RH30 viable cells
100
80
60
40
20
0
UT
IFN-1
Poly(I:C)
IFN-1 +Poly(I:C)
E
% of RD viable cells
100
80
60
40
20
0
ns
*
*
UT
Poly(I:C)
UT
Poly(I:C)
Ctrl-RD
TLR3-RD
F
RD
MW (kDa)
Ctl
Transf.
160
130
100
70
55
36
TLR3-CFP
TLR3 FL
TLR3-NG
TLR3 cleaved
*
GAPDH

Supplementary Table 1 – Cutoffs used for the survival analyses

Figure	Dataset	Tumor	TLR3 Cutoff	Cutoff method	Log2 normalised expression
1B	Missiaglia	FP-RMS	2,659058	Maxstat	Yes
1C	Kuijjer	Osteosarcoma	176,7	KaplanScan	No
S1A	Khan	FP-RMS	7,262	Maxstat	Yes
S1B	Missiaglia	FN-RMS	3,427901	Maxstat	Yes
S1C	Dirksen	Ewing Sarcoma	31,8	KaplanScan	No
S1C	TCGA 2022-v32 Sarcoma	Leiomyosarcoma	1,95	Maxstat	Yes
