# Supplementary material for: The TLR3 L412F polymorphism prevents TLR3-mediated tumor cell death induction in pediatric sarcomas
Source: Cell Death Discov. 2023 Jul 7;9:230. doi: 10.1038/s41420-023-01513-y (PMC10326074; doi:10.1038/s41420-023-01513-y)

Full and uncropped western blot for Figure 2A
Lanes 1, 2 are on the figure

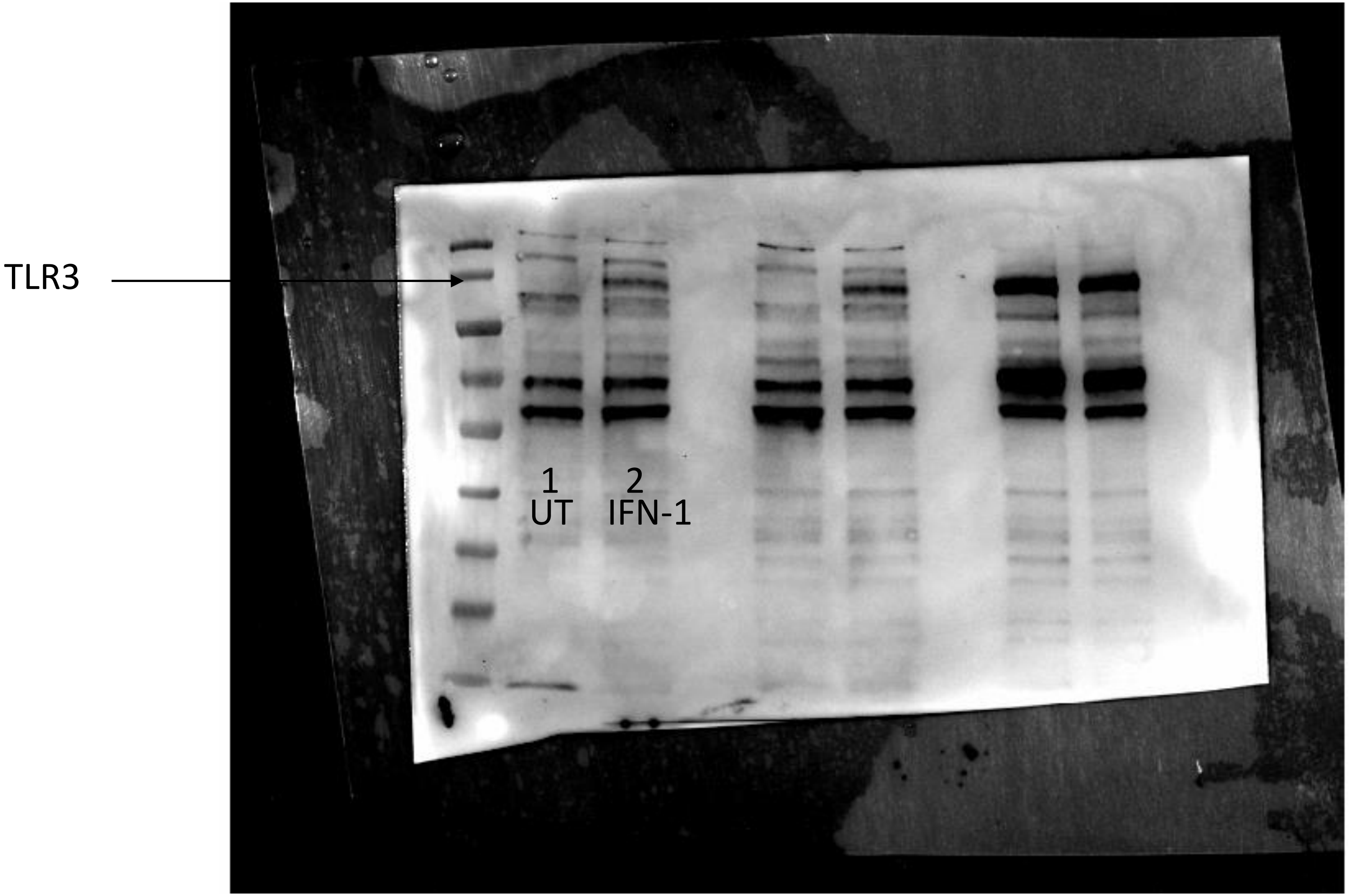

Full and uncropped western blot for Figure 2A
Lanes 1, 2 are on the figure

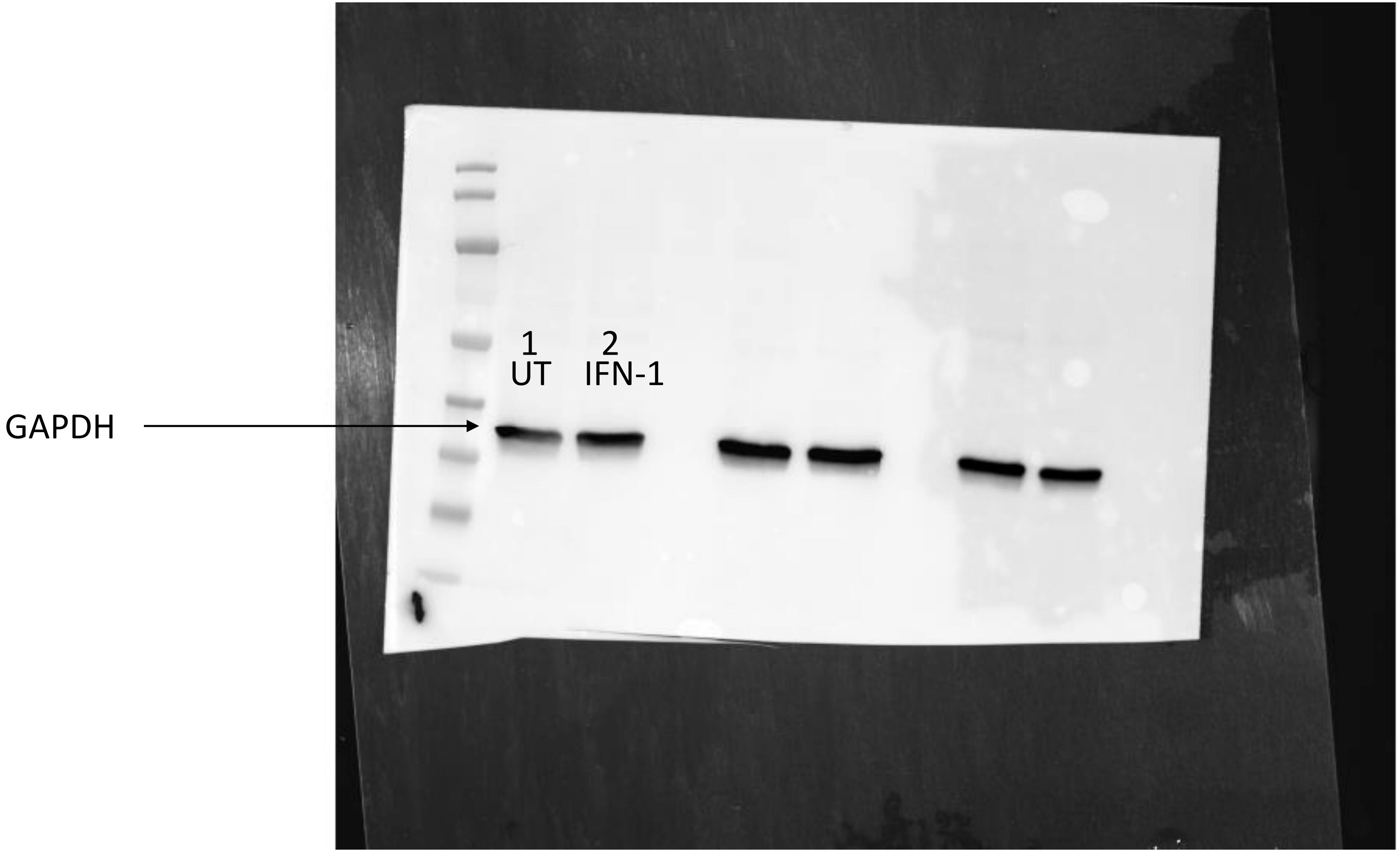

Full and uncropped western blot for Figure S2A
Lanes 1, 2 are on the figure

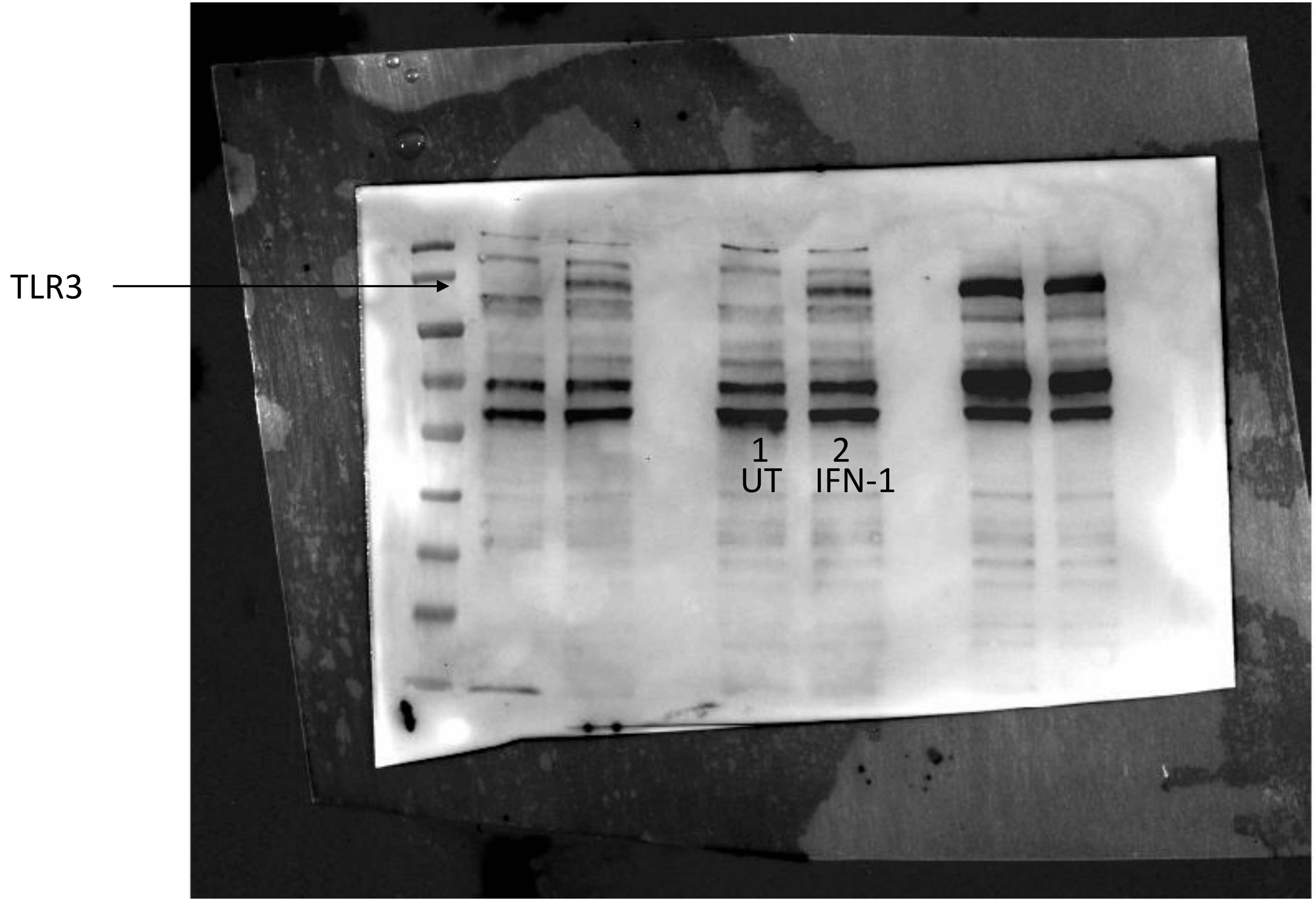

Full and uncropped western blot for Figure S2A
Lanes 1, 2 are on the figure

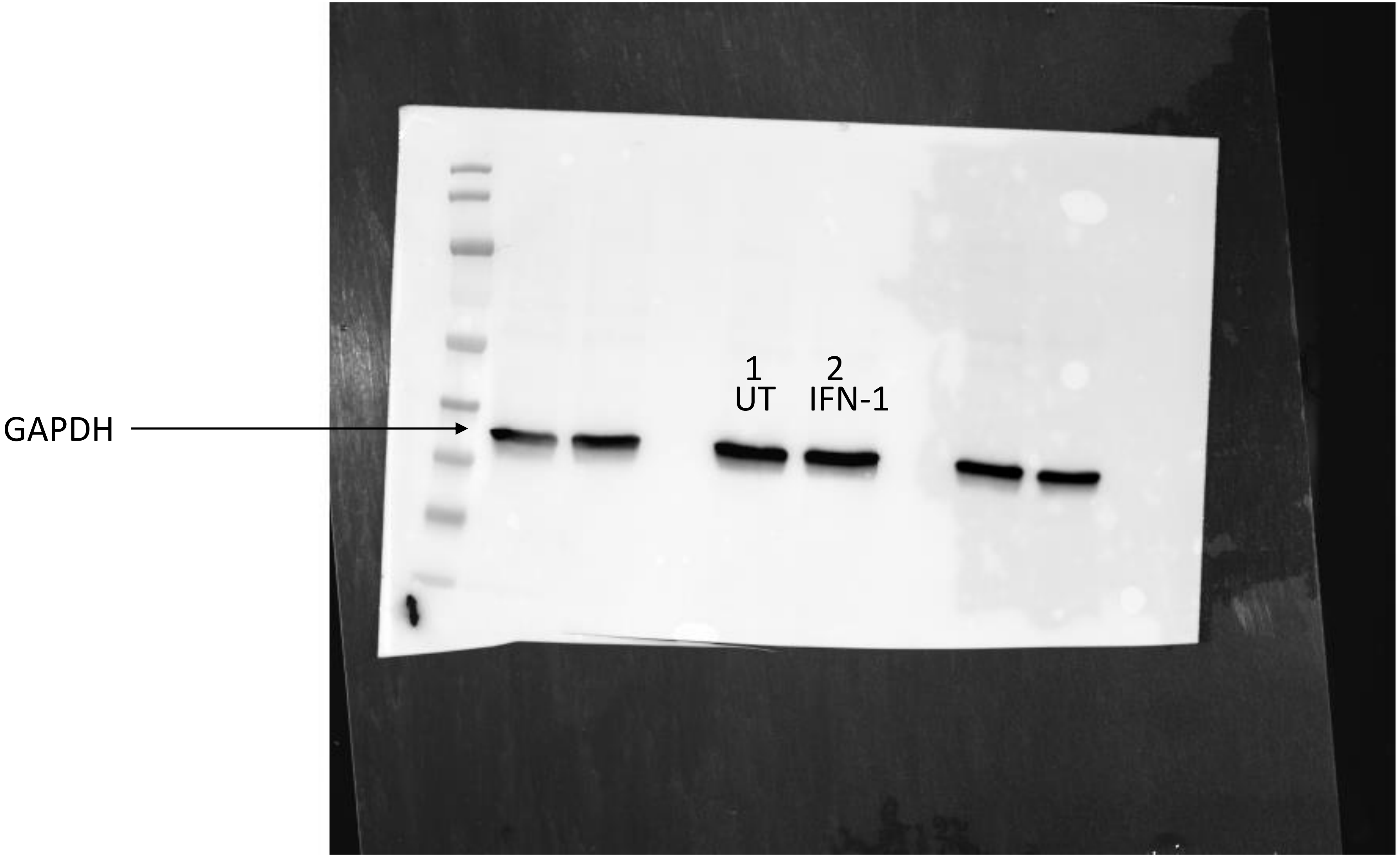

Full and uncropped western blot for Figure S2B
Lanes 1, 2 are on the figure

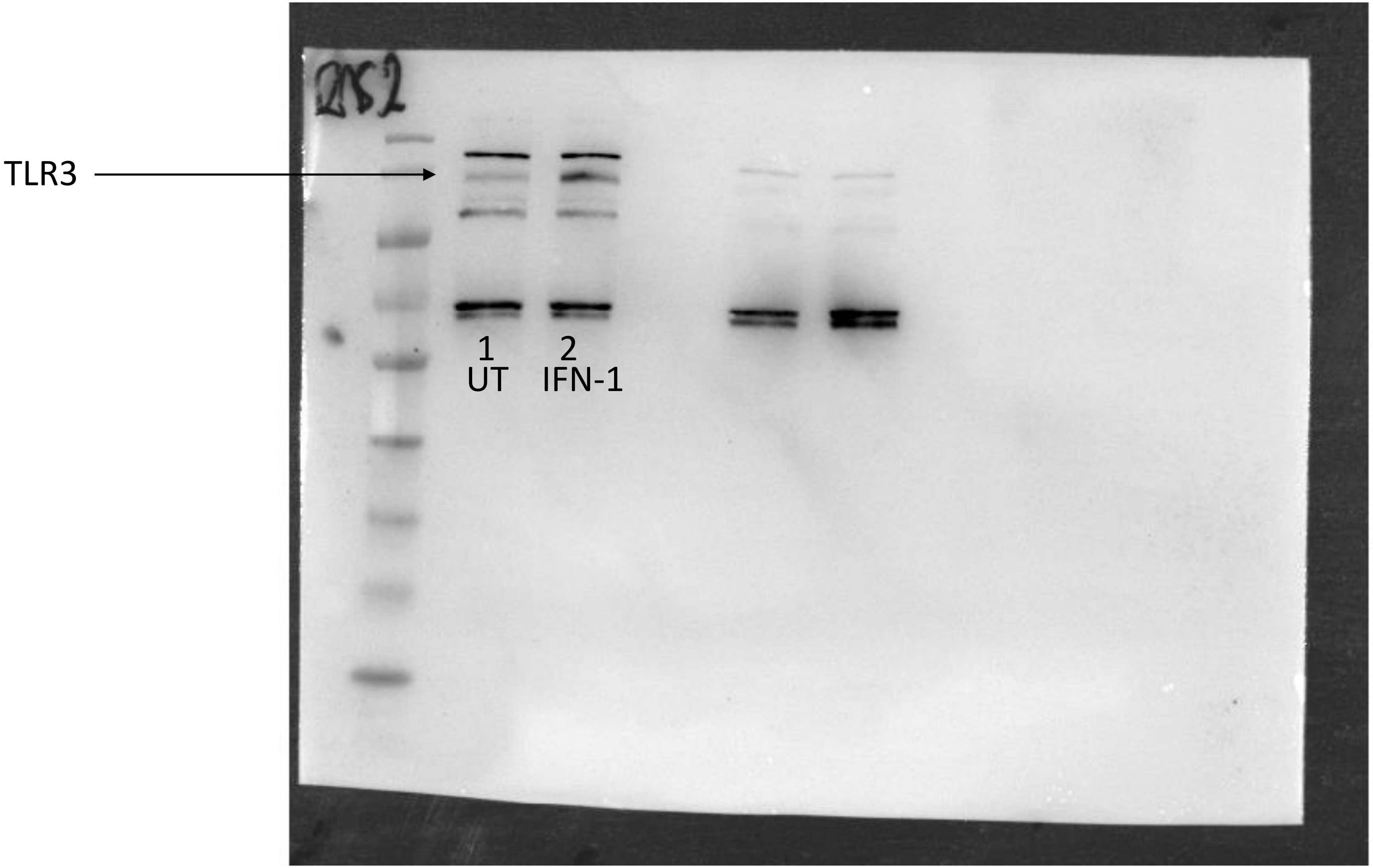

Full and uncropped western blot for Figure S2B
Lanes 1, 2 are on the figure

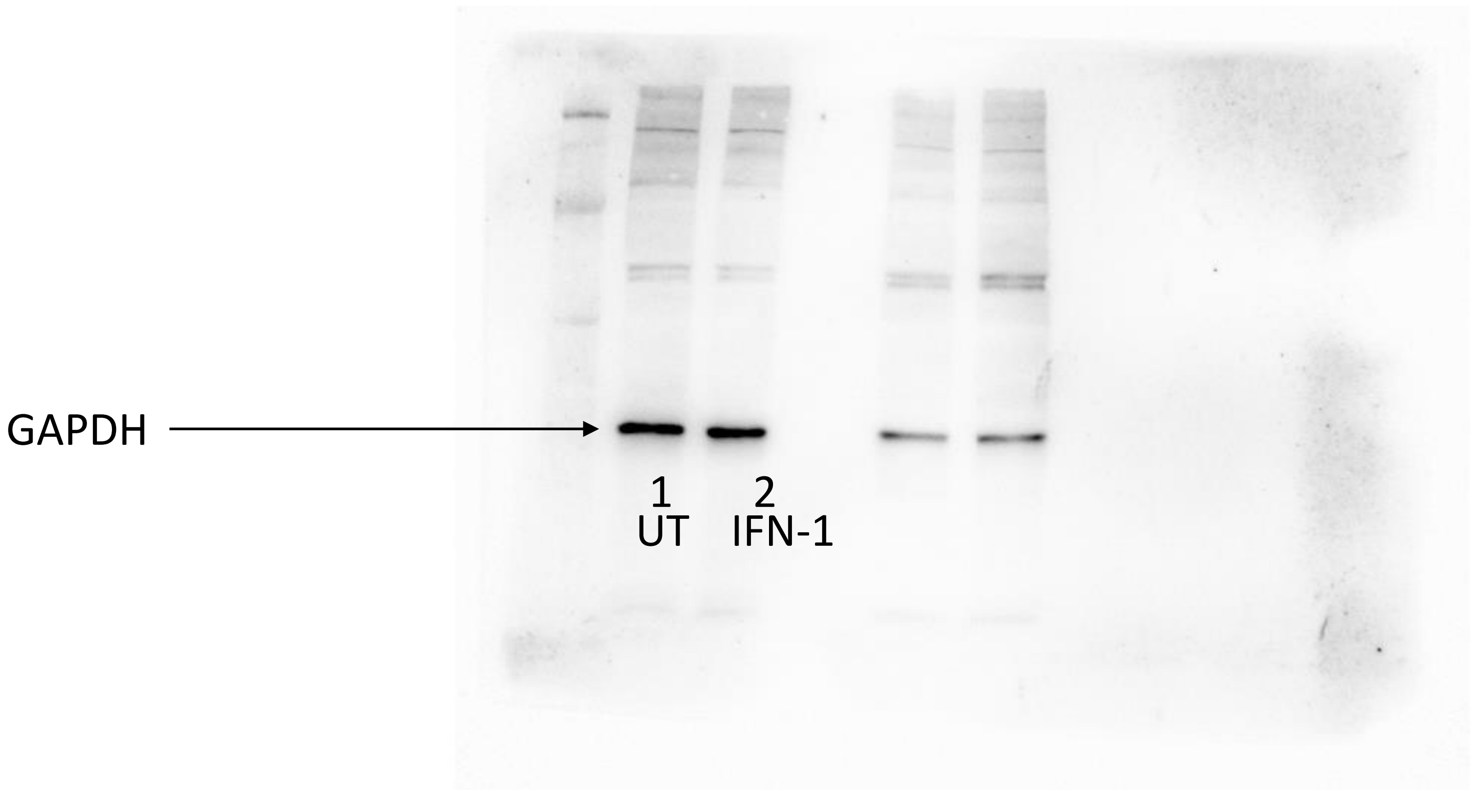

Full and uncropped western blot for Figure S3A
Lanes 1, 2 are on the figure

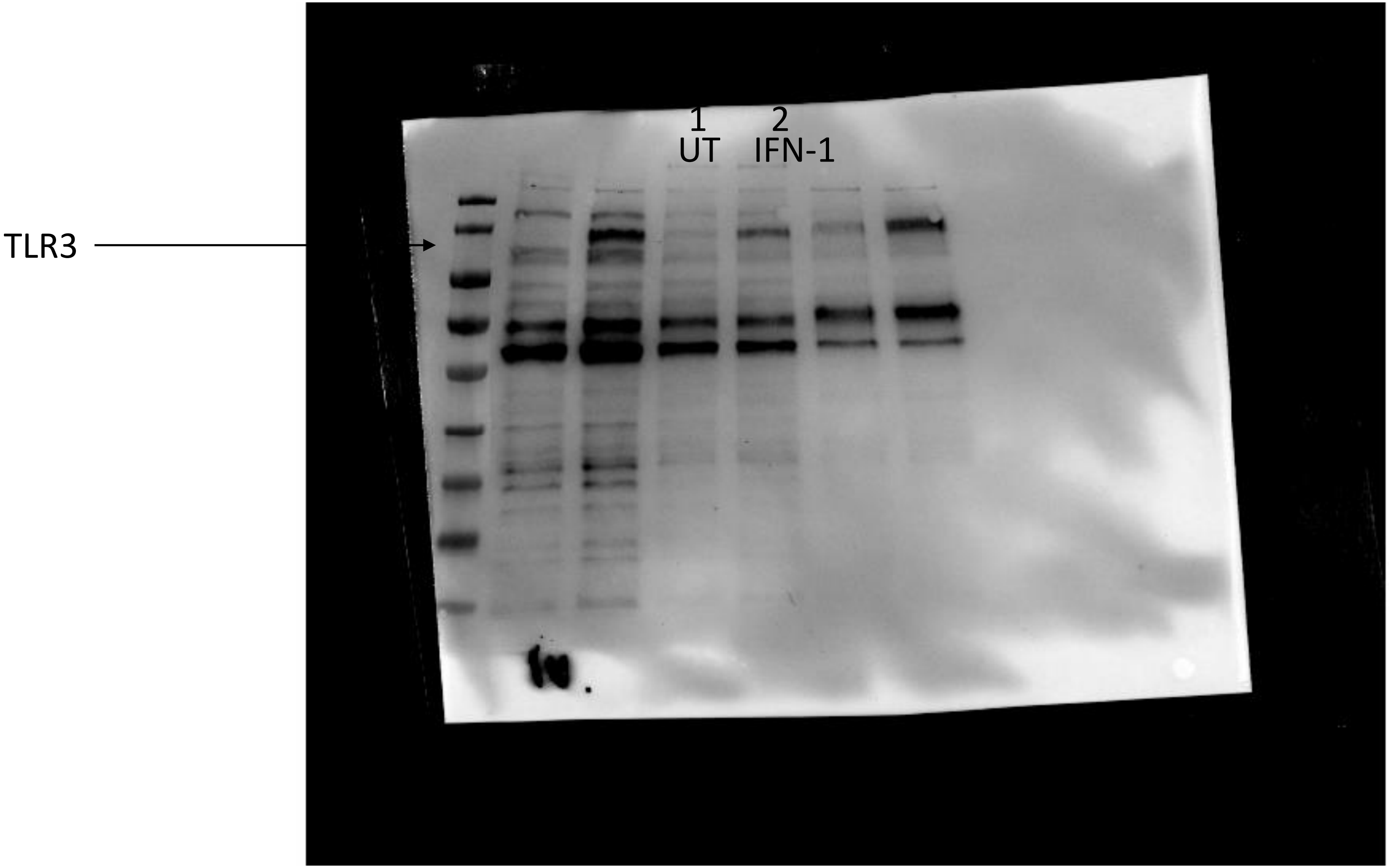

Full and uncropped western blot for Figure S3A
Lanes 1, 2 are on the figure

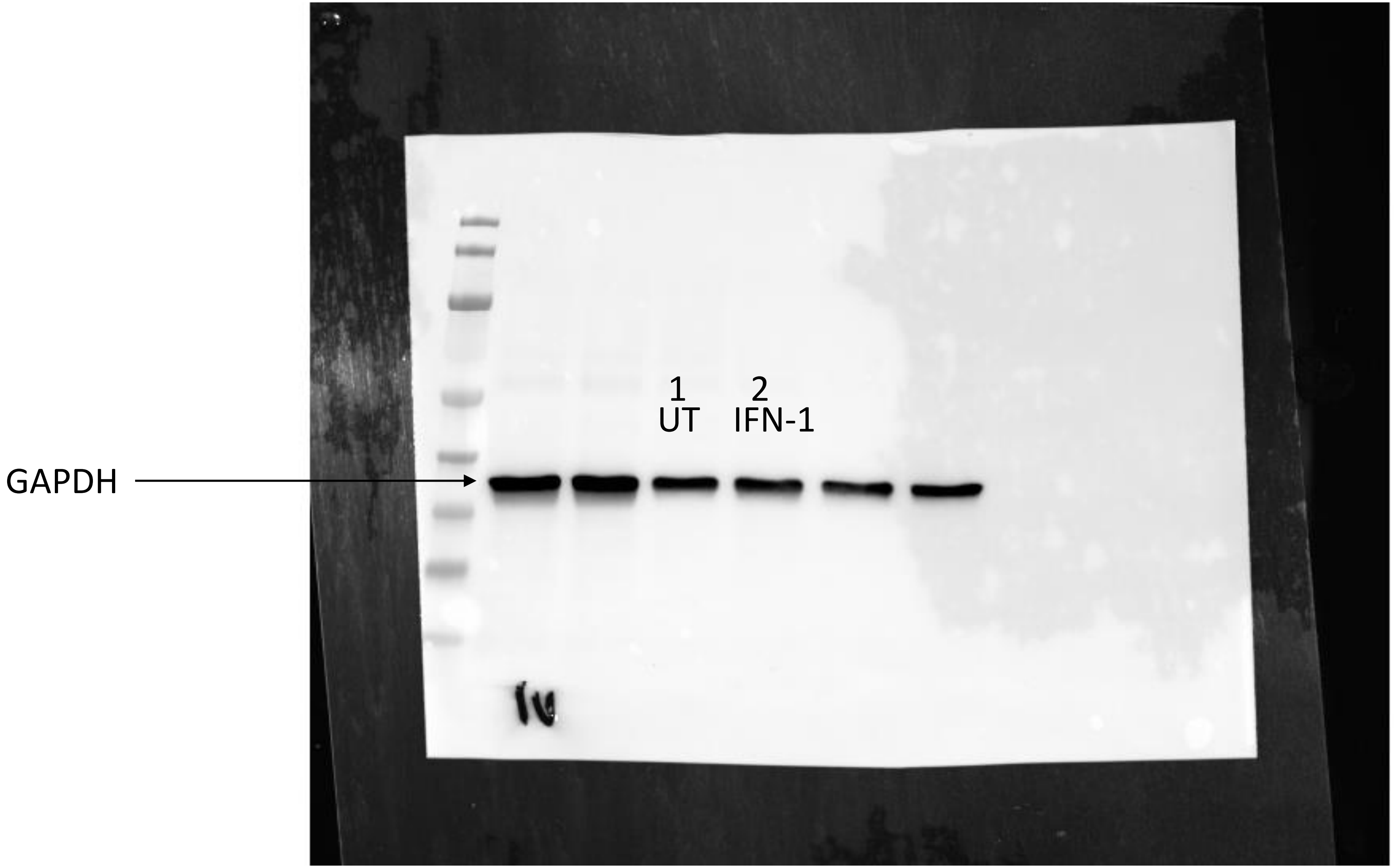

Full and uncropped western blot for Figure S3C
Lanes 1, 2 are on the figure

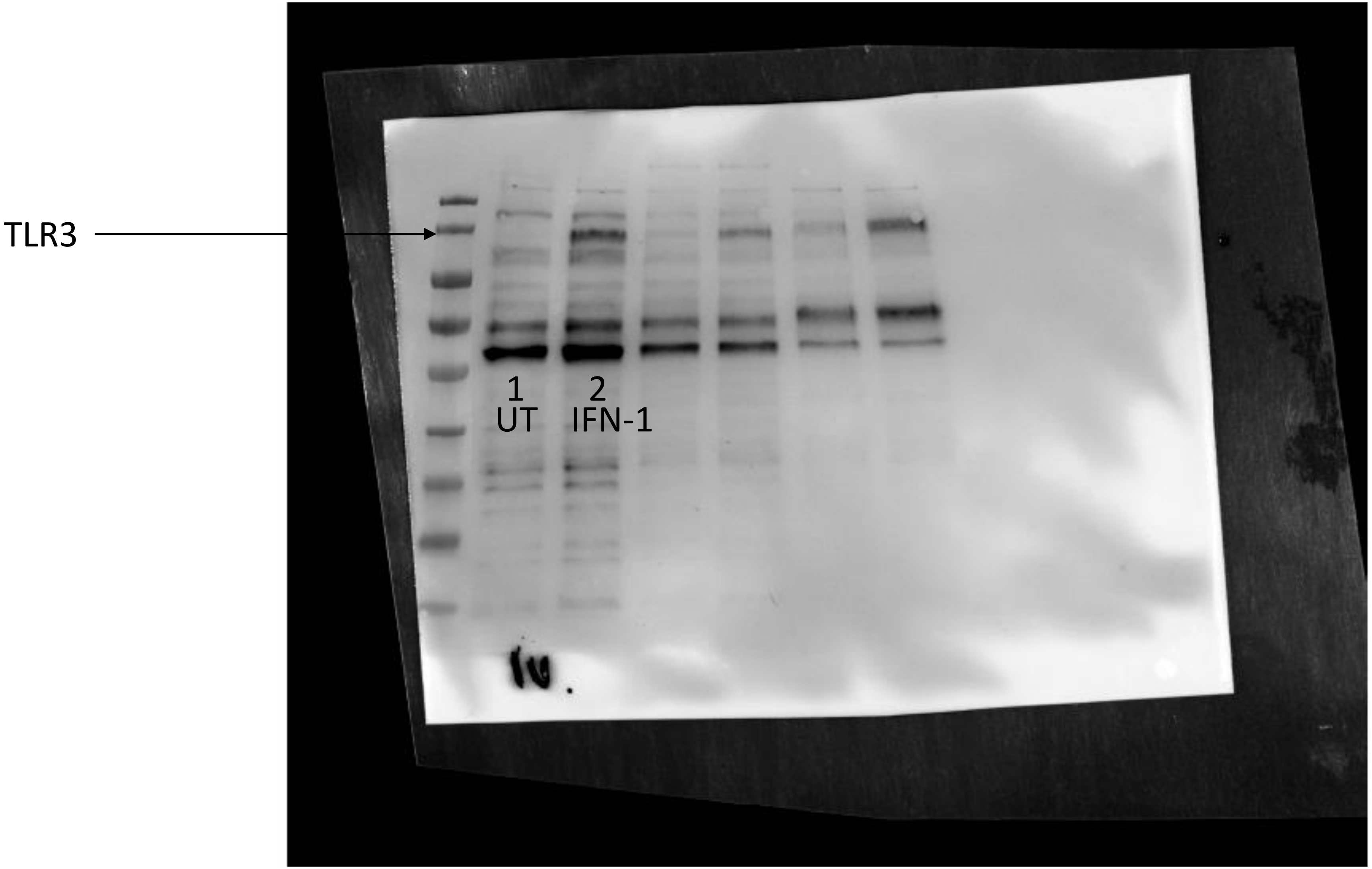

Full and uncropped western blot for Figure S3C
Lanes 1, 2 are on the figure

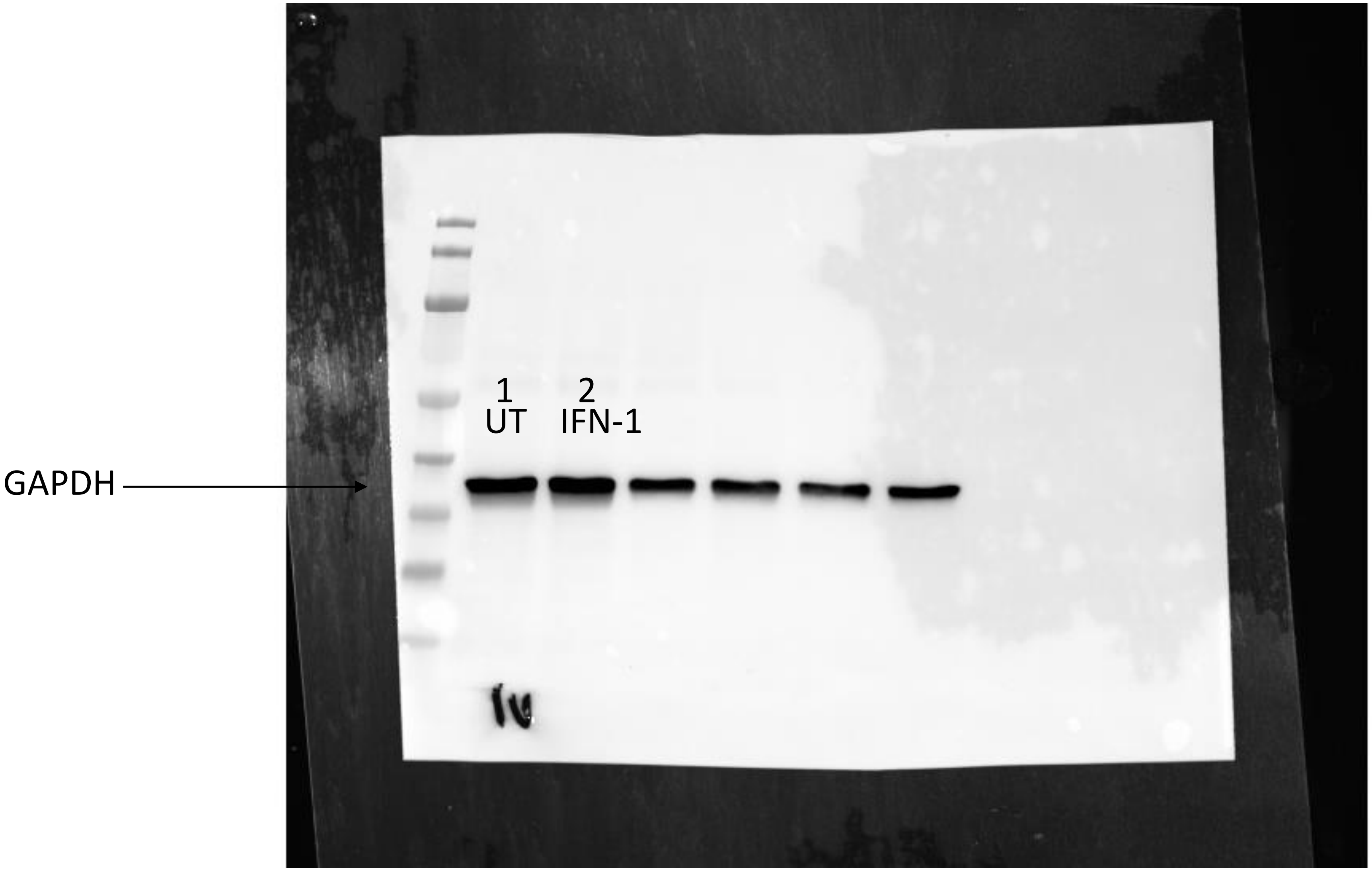

GAPDH

Full and uncropped western blot for Figure S3F Lanes 1, 2 are on the figure

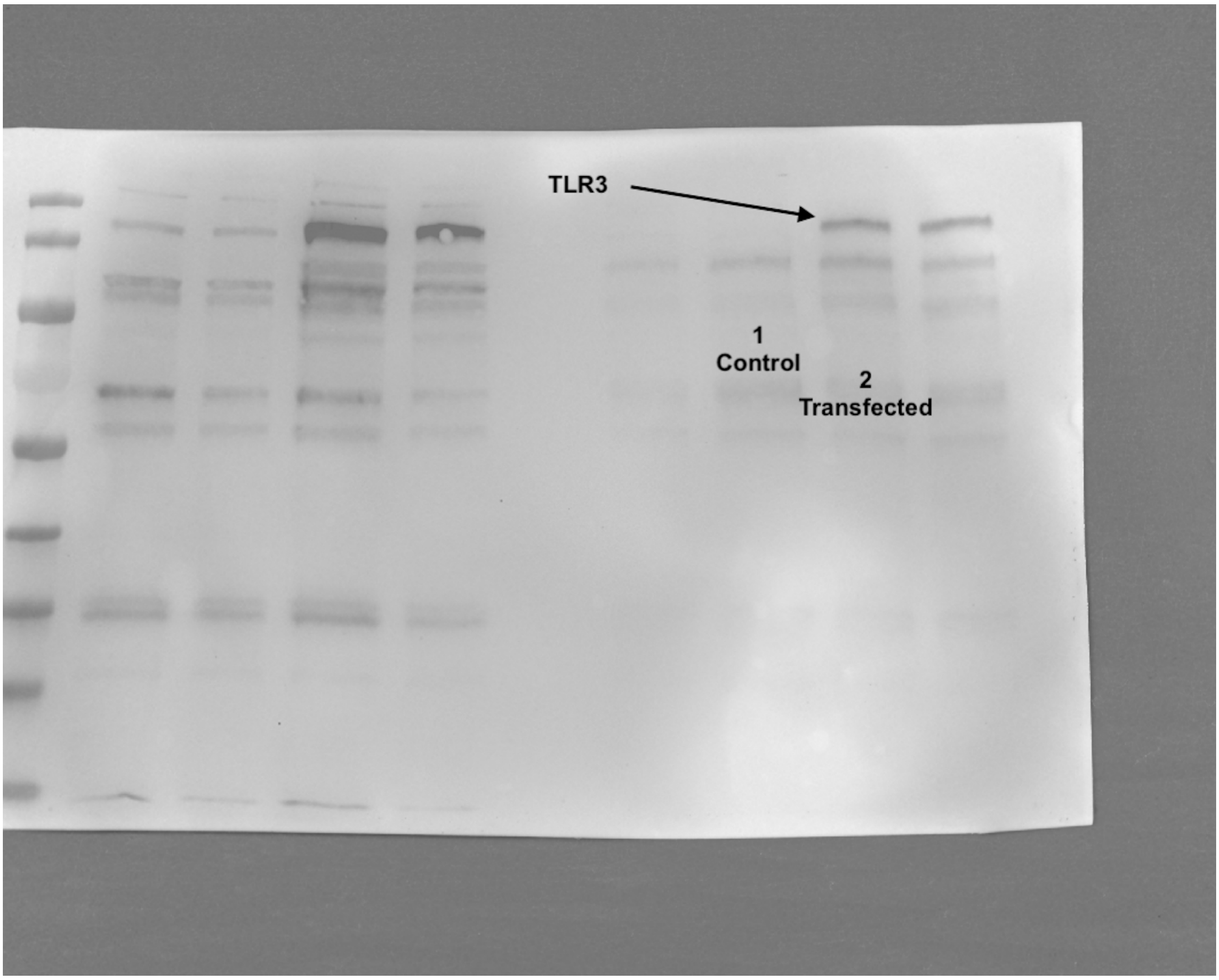

Full and uncropped western blot for Figure S3F
Lanes 1, 2 are on the figure

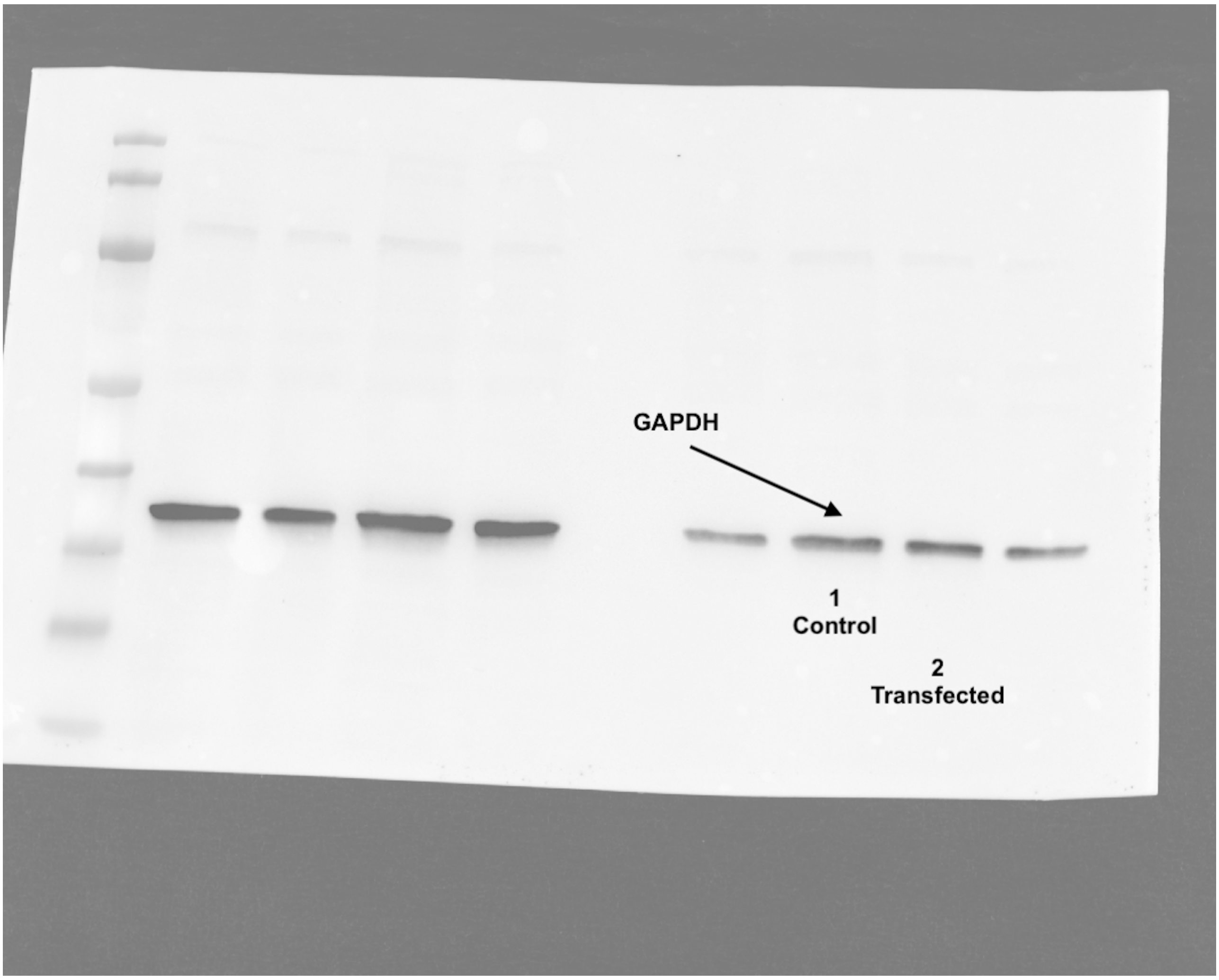

Supplement: Supplementary file 3 — Original Data File [file 41420_2023_1513_MOESM3_ESM.pdf]
